# Editorial: Comprehensive mechanism of edible fungus polysaccharide in immunomodulatory, anti-inflammatory, and hypoglycaemic effect

**DOI:** 10.3389/fnut.2023.1305559

**Published:** 2023-10-19

**Authors:** Changyang Ma, Zhenhua Liu, Shiming Li, Fatma M. El-Demerdash

**Affiliations:** ^1^National Research and Development Center for Edible Fungus Processing Technology, Henan University, Kaifeng, China; ^2^College of Agriculture, Henan University, Kaifeng, China; ^3^Joint International Research Laboratory of Food and Medicine Resource Function of Henan Province, Kaifeng, China; ^4^Department of Environmental Studies, Institute of Graduate Studies and Research, Alexandria University, Alexandria, Egypt

**Keywords:** edible fungus polysaccharides, immunomodulatory effect, inhibition of inflammation, hypoglycaemic effects, anti-aging

Edible fungi have been used as food and herbal medicine for centuries all over the world, particularly in China and several other Southeast Asia countries. Recently, it has been attracting great attentions for its multiple components and various nutritional and medicinal values on health-promoting effects. Polysaccharide is one of the major components in edible fungus and a growing number of researches has been performed on their bioactivities, such as antioxidant, anti-tumor, immunomodulatory, anti-inflammatory, hypoglycaemic effect, and relieving constipation among others. However, more attention should be paid for extensive and detailed research in fungus polysaccharides to overcome difficulties in elucidating the complicated chemical structures of these polysaccharides, determining their corresponding biological activities and underlying mechanisms, and establishing the structure-activity relationship (SAR) (1).

The function of polysaccharides from edible fungus varies by different species and origins, different extraction, separation and purification methods, as well as chemical modifications. Fundamentally, the bioactivities of polysaccharides relay on the characteristics of their molecular structure, including monosaccharide composition, molecular weight (*M*w), chain length, functional groups, glycosidic linkage, branching degree, spatial structure, configuration and others. For example, Yin et al. (1) reviewed number of edible fungus polysaccharides with immunomodulatory activity and found that mushroom β-glucans with *M*w over 10^4^ Da are more active comparing to that with low *Mw*. Recently, Liu et al. reported that a β-glucan from *Grifola frondose* polysaccharides from ultrasonic-assisted extraction could relieve the inflammation response in oxazolone (OXZ)-induced ulcerative colitis (UC) mice, by decreasing the pro-inflammatory cytokines, such as interleukin-1β (IL-1β), tumor necrosis factor α (TNF-α) and increasing interleukin-10 (IL-10). Qiao et al. isolated one pyran polysaccharide with *M*w 3.2 × 10^5^ Da from *Sparassis latifolia* and found that it could promote the proliferation of RAW264.7 and secretion of pro-inflammation cytokines through MyD88-dependent and -independent signaling pathways mediated by toll-like receptor 4 (TLR4). The polysaccharide from *Heimioporus retisporus* with an average *M*w of 1.95 × 10^6^ Da by Feng et al. has shown a significant hypoglycemic effect in a streptozotocin (STZ)-induced diabetic mouse model.

Meanwhile, many polysaccharides from one species of edible fungus could also demonstrate multiple or opposite bioactivities. It has been known that edible fungus polysaccharides, even mutiple polysaccharides from only one species of edible fungus, are important resources to prevent immune disorder, including immunodeficient and immunodepressed conditions (2). Yan et al. reviewed the bioactivities of polysaccharides from *Tremella aurantialba*, and found that these polysaccharides might exert immunostimulatory activity *via* TLR4. Glucuronoxylomannan (TAP-3) with *M*w of 6.24 × 10^5^ Da isolated from the fruiting bodies of *T. aurantialba* could significantly stimulate the secretion of RAW 264.7 macrophages, generating nitric oxide (NO), IL-1β and TNF-α, demonstrating remarkable immune enhancing activity. When the molecular weight of TAP-3 decreased to *M*w 5.3 × 10^3^ Da, it could reduce the levels of NO, IL-1β and TNF-α *via* anti-TLR4 and express immunosuppressive effects (3).

Moreover, there are many reported bioactivities of fungus polysaccharides and they often have connections with each other. For instance, chronic inflammation is widely recognized as one of the key processes that alert the immune system (4) and majority of diseases has closely associated with chronic inflammation or immune disorder (5). Hence the comprehensive analysis of different functions of edible fungus polysaccharides is of importance to discover the action mechanism of their bioactivities, in particular, their antioxidant, anti-inflammatory and immunoregulatory activities. According to a report of Yan et al. it has been revealed that *T. aurantialba* polysaccharides has demonstrated effective inhibitory effects on chronic inflammation, thus protected inflammation-related diseases such as aging, cancer, autoimmune, cardiovascular, and metabolic diseases. Therefore, inhibition of chronic inflammation could protect many different types of diseases. Feng et al. found that a water-soluble neutral polysaccharide with a *M*w of 1.95 × 10^6^ Da isolated from *H. retisporus* significantly reduced the blood glucose level of STZ-induced diabetic mouse and heart visceral organ index by downregulating the inflammatory cytokines like IL-6.

As an important source, edible fungus polysaccharides have been attested to possess multiple health-promoting effects. Recently, study on the biological property of edible fungus polysaccharides has become hot spot and drew great attention in the field of functional foods and nutraceuticals. With the assistance of the most advanced state of the art technology such as omics and artificial intelligence, comprehensive analysis and research in the resource, composition, structural information, and bioactivity of fungus polysaccharides is warranted to provide more in-depth and detailed information in the research and product development of functional foods with edible fungus polysaccharides.

## Author contributions

CM: Conceptualization, Writing—original draft, Funding acquisition. ZL: Writing—original draft. SL: Writing—review and editing. FE-D: Writing—review and editing.
